# Only functional localization is faithful localization

**DOI:** 10.15698/mic2014.04.141

**Published:** 2014-04-07

**Authors:** Roland Lill

**Affiliations:** 1 Institut für Zytobiologie und Zytopathologie, Philipps-Universität Marburg, Robert-Koch-Straße 6, 35032 Marburg, Germany.; 2 Max-Planck-Institut für terrestrische Mikrobiologie, Karl-von-Frisch-Str. 10, 35043 Marburg, Germany.; 3 LOEWE Zentrum für Synthetische Mikrobiologie SynMikro, Hans-Meerwein-Str., 35043 Marburg, Germany.

**Keywords:** Mitochondrial intermembrane space, oxidative protein folding, mitochondrial protein import, iron-sulfur protein biogenesis, CIA machinery

 Eukaryotic cells contain numerous compartments in which highly specialized functions are executed. The functional separation of key cellular processes, such as anabolic and catabolic pathways or the synthesis of RNA and proteins, is crucial for the precise coordination of the biological processes as well as for the extraordinary functional capacity and the high specialization of eukaryotic cells. Generation and maintenance of the subcellular compartmentalization requires accurate targeting of the individual constituents to these compartments in order to guarantee the proper cooperative function of proteins and RNA in the desired locations, yet not in other places of the cell. Elucidating the mechanisms underlying these trafficking and sorting processes has been a main focus of biological research over the past decades, and has identified hundreds of proteins functioning as targeting factors, trafficking chaperones, sorting components, and membrane translocons. Failure to properly localize RNA or proteins to their respective native locale leads to malfunction of the respective cellular compartment resulting in defective cell homeostasis, eventually even causing cell death. Mislocalization of cellular constituents frequently is associated with human disease. Most prominent cases are certain types of lysosomal storage diseases in which acidic hydrolases are mistargeted to the extracellular space leading to an accumulation of undigested material inside lysosomes. Likewise, a trafficking failure of a mutant version of cystic fibrosis transmembrane regulator CFTR retains the protein mainly in the secretory pathway leading to functional deficiency at its native location, i.e. the plasma membrane.

Numerous studies have revealed that a high number of cellular constituents are located in more than one cell compartment. Most prominently this is the case during the dynamic shuttling of both RNA and proteins between the nucleus and cytosol during the various stages of gene expression and protein synthesis. Specialized export and import components assist the nucleo-cytoplasmic trafficking of, e.g., ribosomal proteins, transcription factors and gene regulatory components to assure proper synthesis and localization of cellular RNA and protein compounds. Up to a third of the cellular proteins are located in more than one compartment even without dynamic shuttling, and some of them appear to fulfill their particular function in both locations 1. This suggests that the cell makes efficient use of a single protein’s function by targeting it to various locations. Examples of such proteins are aconitase and fumarase functioning in both mitochondria and the cytosolic-nuclear compartments; tRNA synthetases which aminoacylate their targets in both mitochondria and cytosol; cytochromes P_450_ and cytochrome* b_5_*reductase which operate in both mitochondria and the endoplasmic reticulum. Numerous cases have been published where a particular protein is located predominantly in one compartment but is additionally present in small amounts at other places. This ‘eclipsed’ protein distribution immediately raises the question of whether the secondary, minor localization is functionally relevant or whether it is the result of inaccurate or inefficient targeting mechanisms 2. Another problem concerning secondary protein localization may be a technical and not a biological one. To faithfully detect proteins, researchers are frequently forced to use overexpressed and/or epitope-tagged proteins. Both the surplus of proteins and alterations in the overall structure caused by tagging might result in erroneous subcellular targeting with no functional relevance. In light of these difficulties, the gold standard for determining the biological meaning of a potential second cellular domicile therefore is "functional localization", i.e. the assignment of a particular function of the protein of interest in this locale. However, this approach is usually demanding, as functional assays might not be available or not specific for the considered subcellular compartment. An interesting example is the cysteine desulfurase Nfs1 which is predominantly located in mitochondria where it participates in cellular iron-sulfur (Fe/S) protein biogenesis 3,4. Small amounts, however, have been detected outside mitochondria 5, yet its function does not appear to be in cytosolic Fe/S protein biogenesis 6,7. While the precise function of extra-mitochondrial Nfs1 remains a matter of debate 8,9,10, yeast genetic studies have elegantly shown that this version of Nfs1 is essential for cell viability 11, leaving no doubt for the physiological importance of this protein outside mitochondria.

Dual subcellular localization has recently been suggested for another member of the Fe/S protein biogenesis system, namely Dre2 (in humans also termed anamorsin or CIAPIN1) 12. The protein is an essential part of the cytosolic iron-sulfur protein assembly (CIA) machinery, and together with its intimate partner, the diflavin oxidoreductase Tah18, forms an electron transfer chain that is crucial for the assembly of cytosolic and nuclear Fe/S proteins 13,14. Dre2 itself is a Fe/S protein and was suggested to contain two Fe/S clusters in its C-terminal domain which harbors two conserved cysteine-containing motifs 12. Upon discovery of Dre2, Zhang and colleagues noted that a small amount of epitope-tagged Dre2 was associated with isolated mitochondria. Since the protein was largely protease-resistant, it was suggested to be located in the intermembrane space. Later studies have taken this suggestion a step further to provide compelling *in vitro* evidence for the Mia40-dependent import of the human version of Dre2 (anamorsin) into the intermembrane space 15,16. The extreme C-terminal cysteine residues of anamorsin were shown to be oxidized in a Mia40-dependent fashion and a direct protein interaction was documented between anamorsin and Mia40. Together, these data were taken to suggest that anamorsin is the first imported Fe/S protein of the intermembrane space with a potential function in Fe/S protein biogenesis in this compartment. Notably, the mitochondrial intermembrane space does not contain any known Fe/S protein biogenesis factors, suggesting that Fe/S cluster insertion into anamorsin either may occur spontaneously or may be assisted by yet to be identified factors. Another potential Fe/S protein of this compartment is Mia40 itself, as an overexpressed version of this protein was shown *in vivo* to bind iron 17. Since Mia40 assembles a Fe/S cluster *in vitro*, it was speculated that Mia40 may coordinate a Fe/S cluster in the intermembrane space. However, verification of the presence of such a cofactor on Mia40 *in vivo* is pending.

In an elegant biochemical effort the proteome of the yeast mitochondrial intermembrane space has been identified 18. Conspicuously, Dre2 was not contained in this list, which simply may be due to the fact that minor constituents can easily be missed in such systematic approaches. Hence, it required a dedicated study to reinvestigate the potential localization of Dre2 in this compartment. In lieu of a true functional assay for Dre2 in the intermembrane space, Peleh *et al. *decided to use two major strategies to clarify the issue 19. First, they carefully re-analyzed the specific subcellular localization of Dre2. They indeed find (overproduced) Dre2 located in the cytosol and associated with mitochondria. From a number of technical approaches including protease protection assays and sub-mitochondrial fractionation the authors convincingly conclude that the protein is located outside rather than inside the organelle. The mitochondrial surface-bound fraction of Dre2 is protease-resistant as noted earlier, yet can be released by high salt.

In the second approach, Peleh *et al*. investigated whether yeast Dre2 can use the Mia40 import pathway into the intermembrane space. The first indication that this may not be the case came from yeast Mia40 depletion experiments. Efficient down-regulation of Mia40 did not affect the amount of Dre2 associated with isolated mitochondria, a finding consistent with Dre2 sticking to the outer face of mitochondria, yet inconsistent with a role of Mia40 in Dre2 import into the intermembrane space. Moreover, the C-terminal cysteine residues of Dre2 remained reduced upon Mia40 depletion providing no indication that this oxidoreductase has any influence on the amount and oxidation state of Dre2. Together, these results rendered it unlikely that yeast Dre2 is a bona fide constituent of the mitochondrial intermembrane space.

Peleh *et al.* then go on and take their study beyond a mere protein localization study. They artificially (and quantitatively) direct Dre2 into the intermembrane space by attaching it to the N-terminal targeting information of Mia40 (residues 1-70). The fusion protein (imsDre2) follows the classical presequence-dependent TOM-TIM pathway 20,21, and is hooked up to the inner membrane via the Mia40 prepiece. When imsDre2 was analyzed for its redox status, all its cysteine residues were found to be accessible for modification by alkylating agents indicating that no disulfide bridges were formed upon forced entry into the intermembrane space, nor Fe/S clusters were coordinated. How can this discrepancy to the earlier results 15 be explained? One notable difference may be the source of the protein, yeast Dre2 versus human anamorsin. However, the high conservation of the CIA system in yeast and human cells makes it unlikely that there are major differences between these orthologues 14. A more relevant suggestion has been made by Peleh *et al.* 19. Disulfide bridges could in fact be introduced into imsDre2 *in vitro* during the isolation of mitochondria or upon treatment with the chemical oxidant diamide. However, this oxidation step was not observed, if the redox status of imsDre2 was estimated immediately during cell lysis and not after isolation of mitochondria. From these observations, Peleh *et al.* conclude that there is no indication for any redox modification of the cysteine residues of Dre2 *in vivo* by Mia40 or for the coordination of a Fe/S cluster in the mitochondrial intermembrane space 19. Importantly, this data shows a striking substrate specificity of the Mia40 oxidoreductase for its native substrates typically containing twin CX_3_C or twin CX_9_C motifs 16. In contrast, the twin CX_2_C motif present in the C-terminal domain of Dre2 may not be a native substrate of Mia40. While this finding provides interesting information on the substrate specificity of this oxidoreductase, the results also serve as a fine surrogate for ‘functional localization’ of Dre2, again arguing that *in vivo* the protein not normally enters and is processed in the mitochondrial intermembrane space.

The example of Dre2 provides a paradigm of how other cases of dual localization need to be viewed. The mere presence of a protein in a second compartment should not be regarded as a faithful hint that the protein is functional in this site, the more so if only miniscule amounts are present. Physiological relevance of the secondary localization can be assigned only after establishing the protein’s integration into a biologically relevant process within this compartment.
